# Early Pregnancy Induces Expression of STAT1, OAS1 and CXCL10 in Ovine Spleen

**DOI:** 10.3390/ani9110882

**Published:** 2019-10-30

**Authors:** Yujiao Wang, Xu Han, Leying Zhang, Nan Cao, Lidong Cao, Ling Yang

**Affiliations:** Department of Animal Science, College of Life Sciences and Food Engineering, Hebei University of Engineering, Handan 056021, China; wang60302@126.com (Y.W.); hanxx18832015780@163.com (X.H.); zhangly056000@126.com (L.Z.); m680924250@126.com (N.C.); dong18832072144@163.com (L.C.)

**Keywords:** interferon stimulated genes, pregnancy, spleen, sheep

## Abstract

**Simple Summary:**

Interferon-tau is a maternal recognition factor in ruminants, and spleen plays an essential role in regulating innate and adaptive immune responses. We found that interferon-tau derived from conceptus induces expression of STAT1, OAS1, and CXCL10 in ovine maternal spleen, which may be helpful for maternal immune regulation.

**Abstract:**

Interferon-tau is a maternal recognition factor in ruminant species, and spleen plays an essential role in regulating innate and adaptive immune responses. However, it is not fully understood that early pregnancy induces expression of interferon stimulated genes (ISGs) in the spleen during early pregnancy in ewes. In this study, spleens were collected from ewes at day 16 of the estrous cycle, and on days 13, 16, and 25 of gestation (n = 6 for each group), and RT-qPCR, western blot and immunohistochemistry analysis were used to detect the expression of signal transducer and activator of transcription 1 (STAT1), 2′,5′-oligoadenylate synthetase 1 (OAS1), myxovirusresistance protein 1 (Mx1) and C-X-C motif chemokine 10 (CXCL10). The results revealed that *STAT1*, *OAS1* and *CXCL10* mRNA and proteins were upregulated in the spleens during early pregnancy, and STAT1 protein was located in connective tissue cells in the capsule and trabeculae, and blood cells and lymphocytes in the red pulp. However, early pregnancy had no significant effects on expression of *MX1* mRNA and protein. In conclusion, early pregnancy induces expression of STAT1, OAS1 and CXCL10 in maternal spleen, suggesting that maternal spleen is involved in immune regulation of pregnancy in sheep.

## 1. Introduction

Interferon-tau (IFNT) serves as a key maternal recognition factor in ruminant species, and suppresses endometrial production of prostaglandin F2α via an intrauterine paracrine mechanism [1], to extend the lifespan of ovarian corpus luteum (CL) through a counter-current exchange from the uterine vein to the ovarian artery in sheep [2]. Furthermore, IFNT also exerts systemic effects on maternal physiology to upregulate interferon-stimulated gene 15-kDa protein (ISG15) in the CL and liver through an endocrine manner in sheep [3]. It has been reported that ISG15 [4], 2′,5′-oligoadenylate synthetase 1 (OAS1) [5], myxovirusresistance protein 1 (Mx1) [6] are upregulated in the ovine uterus during early pregnancy. In the extrauterine tissues, such as CL [7], bone marrow [8], thymus [9], spleen [10], and lymph node [11], expression of ISG15 is also increased during early pregnancy in sheep.

There is a wide variety of immune cell population in the spleen that plays an essential role in regulating immune system through blood circulation in mammals [12]. Spleen is an important immune organ, and implicated in regulation of innate and adaptive immune responses [13]. There is a differential expression of protein patterns in the spleen between pregnant and pseudopregnant mice, which are implicated in cell motility and metabolism, suggesting that pregnancy induces changes in the activation state of the splenic lymphocytes [14]. We recently report that three progesterone (P4) receptor isoforms are upregulated in the ovine spleen [15], and expression of cyclooxygenase-2 and PGF synthase are upregulated in splenic trabeculae and splenic cords during early pregnancy [10]. In addition, expression of tumor necrosis factor beta, interleukin (IL)-2, IL-4, IL-5, IL-6 and IL-10 are also upregulated in the maternal spleen during early pregnancy in sheep [16], suggesting that the early pregnancy is involved in regulating expression of genes and proteins in the ovine maternal spleen during early pregnancy.

IFNT and IFN-α have a common receptor in bovine endometrial tissue, and there is no unique IFNT-binding receptor in endometrium [17]. It is through activating Janus kinase (JAK)-signal transducer and activator of transcription (STAT) pathway that IFNT exerts its effects on the bovine endometrium [18]. Expression of interferon stimulated genes (ISGs) is stimulated by IFNT via upregulation of STAT1, STAT2 and IFN regulatory factor-9 in the ovine endometrium during early pregnancy [19]. However, it is unclear that early pregnancy induces expression of ISGs, including STAT1, OAS1, Mx1 and C-X-C motif chemokine 10 (CXCL10) in ovine spleen. In this study, the spleens were sampled from ewes to explore the effects of early pregnancy on expression of ISGs, which may be helpful for making out the formation of maternal immune tolerance and decreasing early embryo loss in ruminants and humans.

## 2. Materials and Methods 

All procedures were approved by the Hebei University of Engineering Animal Care and Use Committee (AEEI-16015).

### 2.1. Animal Tissue Collection

Mature and healthy Small-tail Han ewes (approximately 18 months old) were purchased from Handan Boyuan Animal Husbandry Co., Ltd. (China), and housed under conventional breeding and nutrition level. The ewes with normal estrous cycles were randomly divided into three experimental groups (days 13, 16, and 25 of pregnancy) and a control group of day 16 of the estrous cycle (n = 6 for each group) owing to the P4 and IFNT. After detection of sexual receptivity (day 0 of pregnancy or nonpregnancy) with a vasectomized ram, ewes were bred with intact rams for the three experimental groups, and the nonpregnant ewes were not mated with an intact ram. The ewes were killed, and spleens were sampled on days 13, 16, and 25 after detection of sexual receptivity. Pregnancy was confirmed through observing a conceptus in the uterus. In order to ensure 18 pregnant ewes (three groups), 36 ewes were mated with intact rams. There were 7 mated ewes without pregnancy after killed, and 11 mated ewes were not killed. Ewes from day 16 of the estrous cycle were used as the control group, because the levels of P4 and IFNT are low at this period comparing to other three periods. Splenic samples were immediately fixed in fresh 4% (w/v) paraformaldehyde, and also frozen and stored in liquid nitrogen for following real-time quantitative PCR (RT-qPCR) and western blot analysis.

### 2.2. RNA Extraction and RT-QPCR Assay

Total RNA was isolated from the samples following the manufacturer’s instructions using TRIzol reagent (Invitrogen, Carlsbad, CA, USA), and then treated with DNase (Tiangen Biotech Co., Ltd., Beijing, China). The cDNA was synthesized using a FastQuant RT kit (Tiangen Biotech) following the manufacturer’s instructions. The primer sequences were designed and synthesized by Shanghai Sangon Biotech Co., Ltd. (Shanghai, China) (Table 1). The mRNA expression levels of *STAT1*, *OAS1*, *MX1* and *CXCL10* were analyzed by qPCR using a SuperReal PreMix Plus kit (Tiangen Biotech) according to optimized PCR protocols, and *GAPDH* was amplified in parallels with the ISGs genes. PCR conditions were 40 cycles of 95 °C for 10 s, 57–65 °C (57 °C for *CXCL10*, 60 °C for *OAS1*, 63 °C for *STAT1*, 65 °C for *MX1*) for 20 s, and 72 °C for 25 s. The 2^−ΔΔCt^ analysis method [20] was used to calculate relative expression value with *GAPDH* as a normalization control. The relative expression value from the ewes on day 16 of the estrous cycle was used as normalization control, and set as 1 comparing with that from the three experimental groups.

### 2.3. Western Blot Analysis

The splenic samples were homogenized in RIPA Lysis Buffer (Biosharp, BL504A) with protease inhibitor. The protein concentration was calculated using a BCA Protein Assay kit (Tiangen Biotech), and total proteins were separated by electrophoresis on 12% SDS-PAGE gels. Total proteins were transferred to PVDF membranes (Millipore, Bedford, MA, USA), and then the membranes were blocked with 5% skimmed milk powder. The membranes were incubated with a goat anti-STAT1 polyclonal antibody (Abcam, Cambridge, UK, ab230428, 1:1000), a rabbit anti-OAS1 polyclonal antibody (Abcam, ab86343, 1:1000), a mouse anti-Mx1 monoclonal antibody (Santa Cruz Biotechnology, Santa Cruz, CA, USA, sc-166412, 1:1000), and a mouse anti-CXCL10 monoclonal antibody (Santa Cruz Biotechnology, sc-374092, 1:1000), respectively. After washing, the membranes were incubated with goat anti-mouse IgG-HRP (Biosharp, BL001A) or rabbit anti-goat IgG-HRP (Biosharp, BL004A) with 1:10 000 dilution, and the signals were detected using an ECL western blotting detection reagent (Tiangen Biotech). The immunospecific bands were quantified using Quantity One V452 (Bio-Rad Laboratories, Hercules, CA, USA) with GAPDH as an internal control protein. GAPDH was detected using an anti-GAPDH antibody (Santa Cruz Biotechnology, Inc., sc-20357, 1:1000).

### 2.4. Immunohistochemistry Analysis

The fixed splenic tissues were dehydrated in ethanol, and embedded in paraffin. Splenic tissues were cut to 5 μm-thick sections, and followed by deparaffinization and rehydration. Several sections were stained by hematoxylin and eosin. Endogenous peroxidase activity of the sections was quenched using 3% H_2_O_2_, and nonspecific binding was reduced with 5% normal goat serum. Immunohistochemical localization of STAT1 protein was performed using the goat anti-STAT1 polyclonal antibody (Abcam, ab230428; 1:200), and negative controls were treated with goat IgG at equivalent concentration. The antibody binding sites in the tissue was visualized using a DAB kit (Tiangen Biotech), and then nuclear was stained with hematoxylin. The images were captured using a light microscope (Nikon Eclipse E800, Tokyo, Japan) with a digital camera (AxioCam ERc 5s), and the intensity of staining and density of the stained cells were analyzed through the images. The immunostaining intensity of the different samples from different ewes was rated by 2 different investigators in a blinded fashion, and the histological subtypes were analyzed by assigning an immunoreactive intensity of a scale of 0 to 3, as described previously [10]. An intensity of 3+ was given to the cells with the highest staining intensity, and an intensity of 0 was assigned to cells with no immunoreactivity.

### 2.5. Statistical Analysis

Data for relative expression levels of *STAT1*, *OAS1*, *MX1* and *CXCL10* mRNA and proteins were analyzed using a completely randomized design with six animals per group via the Proc MIXED procedure in SAS (Version 9.1; SAS Institute, Cary, NC, USA). Duncan method was used to compare the relative expression levels of the different groups as described previously with ISGs instead of ISG15 and prostaglandin synthases [10]. Data are presented as least squares means. *p* < 0.05 was considered significantly different. 

## 3. Results

### 3.1. Expression of STAT1, OAS1, MX1 and CXCL10 mRNA in the Spleens

**Figure 1:**
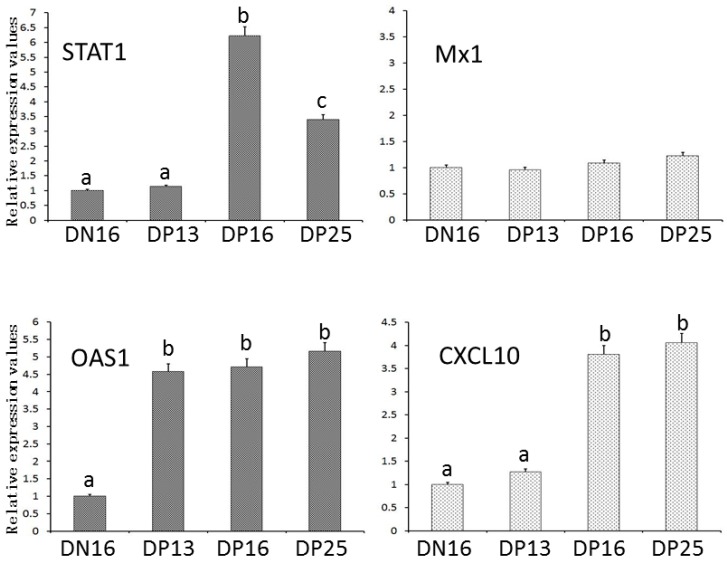
Relative expression values of *STAT1*, *OAS1*, *MX1* and *CXCL10* mRNA in ovine spleens measured by real-time quantitative PCR. Note: DN16 = Day 16 of the estrous cycle; DP13 = Day 13 of pregnancy; DP16 = Day 16 of pregnancy; DP25 = Day 25 of pregnancy. Significant differences (*p* < 0.05) are indicated by different letters within same color column.

Figure 1 showed that the relative expression levels of *STAT1* and *CXCL10* mRNA were upregulated in the spleens at days 16 and 25 of pregnancy comparing with that at day 16 of the estrous cycle and day 13 of pregnancy (*p* < 0.05). The relative expression level of *OAS1* mRNA was higher during early pregnancy than that at day 16 of the estrous cycle (*p* < 0.05). Furthermore, there was no significant difference in expression of *MX1* mRNA among the four groups (*p* > 0.05).

### 3.2. Expression of STAT1, OAS1, Mx1 and CXCL10 Proteins in the Spleens

**Figure 2:**
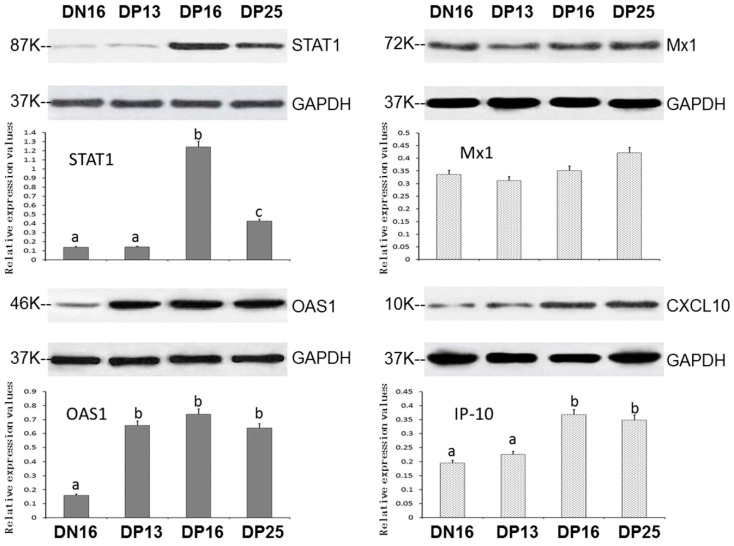
Expression of STAT1, OAS1, Mx1 and CXCL10 proteins in ovine spleens analyzed by western blot. Note: DN16 = Day 16 of the estrous cycle; DP13 = Day 13 of pregnancy; DP16 = Day 16 of pregnancy; DP25 = Day 25 of pregnancy. Significant differences (*p* < 0.05) are indicated by different superscript letters within the same color column.

It was revealed in Figure 2 that there was an upregulation of STAT1 and CXCL10 proteins on days 16 and 25 of pregnancy (*p* < 0.05), and early pregnancy induced upregulation of OAS1 proteins in the spleens (*p* < 0.05). However, expression of Mx1 protein was independent on pregnant status and pregnant period (*p* > 0.05).

### 3.3. Immunohistochemistry for STAT1 Protein in the Spleens

**Figure 3:**
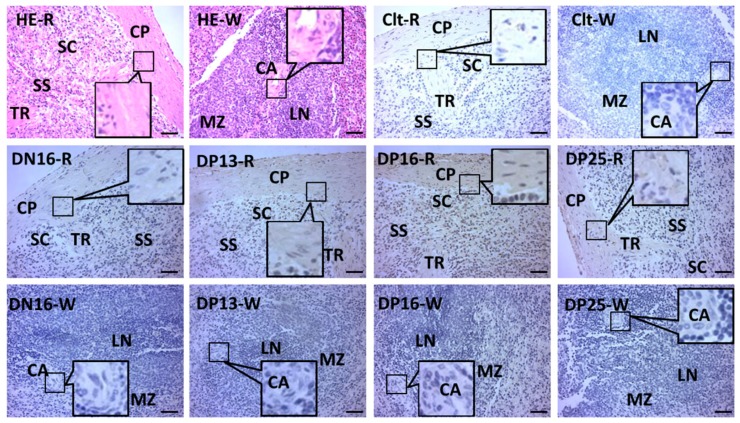
Representative immunohistochemical localization of STAT1 protein in ovine spleens. The spleen is divided into red pulp (R) and white pulp (W), and surrounded by a thickened capsule. Capsule (CP) with several trabeculae (TR) projects into the substance of the spleen. Note: HE = stained by hematoxylin and eosin; SS = splenic sinuses; SC = splenic cords; MZ = marginal zone; LN = lymphoid nodule; DN16 = day 16 of the estrous cycle; DP13 = day 13 of pregnancy; DP16 = day 16 of pregnancy; DP25 = day 25 of pregnancy. Bar = 50 µm.

In the Figure 3, STAT1 was located in cytoplasm of connective tissue cells in the capsule and trabeculae, and blood cells and lymphocytes in the red pulp. The staining intensity for STAT1 protein in the splenic samples were 0, 1+, 1+, 3+, and 1+ for the negative control, the spleens from day 16 of the estrous cycle, and spleens from days 13, 16, and 25 of pregnancy (Figure 3).

## 4. Discussion

The size and number of macrophages of maternal spleen enhances during pregnancy in mice [21,22], and spleen is implicated in maternal innate and adaptive immune responses [13]. In this study, early pregnancy induced upregulation of *STAT1* mRNA and protein in the maternal spleen. It is mainly through activating STAT1 that type I interferon exerts its effects on spleen via recruitment of neutrophils in mice [23]. IFNT stimulates expression of endometrial STAT1 and STAT2 through JAK-STAT pathway in sheep and cattle [18,19], which affect DNA binding, and transcriptional activation [24]. Immune responses in diffuse large B-cell lymphoma are related to the IFN-γ-STAT1- Interferon regulatory factor 1 axis [25]. JAK-STAT pathway participates in innate and adaptive immunities through regulating development of immune system and fate of T helper cells [26]. Therefore, the upregulation of STAT1 in maternal spleen may be involved in regulating splenic immune response during early pregnancy in sheep.

Our results revealed that early pregnancy induced upregulation of *OAS1* mRNA and protein in the maternal spleen. Expression of *OAS1* mRNA is upregulated in bovine blood neutrophils, with a peak around days 18 to 21 of pregnancy [27]. A viable conceptus enhances expression of *OAS1* mRNA in peripheral blood mononuclear cells (PBMCs) between days 15 and 22 post-timed artificial insemination in cows [28]. *OAS1* mRNA value is increased in endometrium, jugular blood and CL from day 15 of pregnant ewes comparing with that from nonpregnant ewes [7]. Early pregnancy stimulates *OAS1* gene expression in hepatic hepatocytes on day 18 of pregnancy, and recombinant bovine IFNT induces *OAS1* gene expression in vitro in bovine hepatocytes [29]. OAS is an interferon-induced antiviral enzyme, and OAS1 silencing leads to downregulation of *IL-1β*, *TNF-α* and monocyte chemoattractant protein-1 in THP-1 cells [30]. Therefore, early pregnancy induces upregulation of OAS1 in maternal spleen, which may be helpful for immune regulation of maternal spleen during early pregnancy in sheep.

It was revealed in this study that early pregnancy had almost no effects on the expression of *MX1* mRNA and protein in the maternal spleens. Mx1 is a dynamin-like guanosine triphosphatase that is implicated in the cellular antiviral response through binding to viral nucleoproteins [31]. Bovine conceptus signaling induces upregulation of *MX1* mRNA in peripheral blood leukocytes (PBLs) on days 18 and 20 of pregnancy [32]. Expression of *MX1* mRNA is upregulated in the CL from day 12 to 14 of pregnancy in sheep [33], and Mx1 protein level is enhanced in uterine flushes after day 15 pregnancy comparing with that from cyclic ewes via an ‘unconventional’ secretory pathway [34]. *MX1* mRNA is upregulated in the liver on day 18 pregnancy comparing with that in nonpregnant heifers [35]. Bacterial load induces upregulation of *MX* mRNA in liver, but it does not work in Peyer’s patches and spleen, suggesting that induction of *MX1* expression is tissue-selective in mice [36]. Therefore, Mx1 expression in maternal spleen is not affected by pregnancy, suggesting that early pregnancy induces the expression of Mx1 in a tissue-selective manner during early pregnancy in sheep.

It is found that expression of *CXCL10* mRNA and protein was enhanced in ovine maternal spleen during early pregnancy. After treatment with bacterial lipopolysaccharide (LPS) via intravenous injection, *CXCL10* mRNA is upregulated in the splenic red pulp of mice [37]. Expression *CXCL10* mRNA is upregulated in bovine endometrium and PBLs on days 15 and 18 of pregnancy, and can be induced by IFNT in cultured endometrial tissue or PBLs [38,39]. Early pregnancy induces upregulation of *CXCL10* gene in the endometria and PBMCs in sheep [40,41]. *CXCL10* mRNA in endometrium and protein in the uterine flushing are increased from day 14 to 20 of pregnancy, which are involved in regulating endometrial functions through recruitment of immune cells in sheep [42]. There is an upregulation of *CXCL10* mRNA in porcine conceptuses from day 15 to 114 of pregnancy, which is implicated in recruiting immune cells into conceptuses [43]. Therefore, the upregulation of CXCL10 may participate in the regulating maternal splenic functions during early pregnancy in sheep. 

The spleen is involved in lymphocyte recirculation and immune surveillance of the blood, and comprised of red and white pulps [44]. Our immunohistochemistry results revealed that STAT1 protein was located in cytoplasm of the cells in capsule, trabeculae and red pulp (Figure 3). The staining intensity for STAT1 was stronger in the capsule, trabeculae and red pulp from the ewes on day 16 of pregnancy (Figure 3). Macrophages in spleen exert distinct functions according to the localization in the splenic compartment, which are implicated in innate and adaptive immune responses [45]. There are undifferentiated monocytes in the spleen, which assemble in the cords of red pulp. Splenic monocytes exit the spleen en masse, and participate in regulation of inflammation through blood circulation [46]. Therefore, during early pregnancy in sheep, the upregulation of STAT1 in the capsule, trabeculae, and red pulp may participate in regulation of maternal immune tolerance. 

The effects of early pregnancy on the ISGs expression are mainly due to P4 and IFNT in the ovine spleen. IFNT (Protein X) is increased between days 14 and 21 in sheep [47], which is almost similar to the expression pattern of STAT1 in the spleen. In addition, IFNT or early pregnancy induces upregulation of *STAT1* gene in the endometria and PBMCs in sheep [19,41]. Therefore, the upregulation of STAT1 in the spleen may be related with IFNT. The P4 concentration in plasma is significantly higher on days 12 and 13, and lower on days 15–16 during ovine estrous cycle [48]. Furthermore, P4 concentration in plasma is also significantly higher during early pregnancy. The expression of OAS1 and CXCL10 in the spleen was higher during early pregnancy in this study. The expression of *CXCL10* genes in the ovine uterus is regulated by early pregnancy, P4, and IFNT [40], so higher levels of OAS1 and CXCL10 in the spleen may be stimulated by P4 and IFNT. However, CXCL10 is also known as interferon gamma-induced protein 10. Interferon gamma is the type II interferon, while IFNT is the type I interferon, so the expression pattern of CXCL10 is different from other interferon-stimulated genes. Furthermore, expression of Mx1 in the spleen was different from that in ovine uterus and CL during early pregnancy [32,33]. It has been reported that IFNT induces the expression pattern of ISG15-conjugated proteins in a tissue-specific manner [49], so the expression pattern of Mx1 in the spleen may be different from that in other tissues. 

## 5. Conclusions

Early pregnancy induced upregulation of STAT1, OAS1 and CXCL10 in the spleens, but there was no significant effect on expression of Mx1. Furthermore, the STAT1 protein was located in connective tissue cells in the capsule and trabeculae, and blood cells and lymphocytes in the red pulp. Therefore, early pregnancy has effects on expression of STAT1, OAS1 and CXCL10 in maternal spleen, which may be beneficial for regulation of maternal splenic immune response during early pregnancy in sheep.

## Figures and Tables

**Table 1:** Primers used for RT-qPCR.

Gene	Primer	Sequence	Size (bp)	Accession Numbers
*STAT1*	Forward	GTGGCGGAGAGTCTGCAGCA	190	NM_001166203.1
	Reverse	GGTGAGTTGGCATGCAGGGC		
*OAS1*	Forward	AGCCTTCCTGAAGAGTCGTCCTAC	88	XM_012097882.2
	Reverse	TCCAAGCTGCTCCTTACACAGTTG		
*MX1*	Forward	CCACCACCGACAGCTCCCCT	147	NM_001009753.1
	Reverse	GCAGGTGTGGGCGTGAAGCA		
*CXCL10*	Forward	TCTAGGAACACACGCTGCAC	108	NM_001009191.1
	Reverse	GACACGTGGGCAGGATTGAC		
*GAPDH*	Forward	GGGTCATCATCTCTGCACCT	176	NM_001190390.1
	Reverse	GGTCATAAGTCCCTCCACGA		
